# Rare Head and Neck Benign Mesenchymoma in Close Proximity to Submandibular Gland in a Pediatric Patient: Case Report and Review of the Literature

**DOI:** 10.1155/2015/131469

**Published:** 2015-05-12

**Authors:** Priyanka Jain, Shraddha Mukerji

**Affiliations:** ^1^University of Texas Medical Branch, Galveston, TX, USA; ^2^Department of Pediatric Otolaryngology, University of Texas Medical Branch, Galveston, TX, USA

## Abstract

Pediatric head and neck masses are commonly congenital in origin or of infectious etiology. We present a rare case of benign mesenchymoma in close proximity to the submandibular gland in an otherwise asymptomatic child. Computerized tomography (CT) scan of the head and neck area revealed a benign lesion, which was later determined to be a benign mesenchymoma on histopathology. The child did well after surgery without any reported recurrence. We discuss the salient features of a benign mesenchymoma in a child and also discuss relevant imaging and management.

## 1. Introduction

Pediatric head and neck masses are often congenital in origin or may be associated with infection. The diagnosis and management of such lesions are usually straightforward. Uncommonly, some patients develop unusual masses that require further workup and require excision biopsy for a definite diagnosis. We present a rare case of a benign, noninfective lesion in close proximity to the submandibular gland in a child. Permission for publication of this case was obtained by the University of Texas Medical Branch Institutional Review Board.

## 2. Case Report

A 9-year-old girl presented to the Outpatient Otolaryngology Department with swelling under the right jaw that had slowly increased in size for the past two years. There was no associated fever, pain, voice changes, breathing difficulty, dysphagia, odynophagia, weight loss, and/or history of exposure to tuberculosis. There was a history of exposure to cats. Exposure to cats with the onset of symptoms in the patient led to cat scratch disease and fungal infections being included in the differential diagnosis. These were ruled out with a negative cat scratch titer and negative toxoplasma IgG.

Physical exam showed the presence of two distinct swellings in the submental and right submandibular (SM) area. The swelling in the right SM area was about 2 × 2 inches and the submental area swelling measured approximately 1.5 × 1.5 inches. The swellings felt firm and nodular and were not freely mobile. The remaining of the ear, nose, and throat and general physical exam was normal. Laboratory tests were negative.

**Figure 1:**
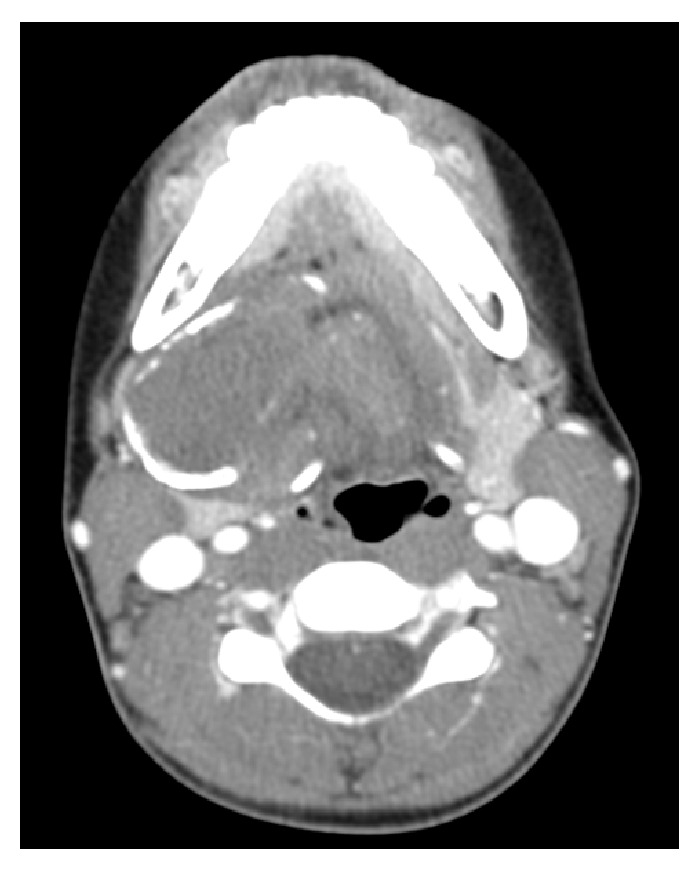
Contrast CT scan showing presence of a heterogeneous, well-defined lesion with peripheral calcification without bone involvement.

Ultrasound (US) of the neck showed the presence of two calcified masses with heterogeneous echotexture. A computerized tomography (CT) scan of the head and neck area with contrast was obtained. A nonenhancing, multilobulated mass with peripheral rim calcification was noted in the SM region mostly on the right side but crossing the midline. The peripheral rim calcifications could signify precursor to bone development within the mass or local necrosis due to mass effect. It measured 4.88 × 3.55 cm (Figure 1). The right SM gland was distinct from the swelling. The remaining salivary glands were normal. The cervical vascular structures were also normal. CT scan characterization of the mass was suggestive of a benign mesenchymal lesion. Other possible differentials included calcified lymph node, calcified plunging ranula without any intraoral component, or liposarcoma.

Surgical excision of the mass was performed through a routine right SM approach. The entire mass was removed along with a surrounding cuff of normal tissue. Since the right submandibular gland was distinct from the swelling and did not seem to be involved at the time of surgery, it was not removed. The mass was a well circumscribed and encapsulated soft tissue lesion with adipose tissue, fibrosis, smooth and skeletal muscle, cartilage, and bone fragments. A thin outer rim was calcified, but the rest of the cut surface was mostly yellowish white with a whorled pattern. The findings were consistent with those of a benign mesenchymoma. The child did well at one- and 6-month follow-up periods without any recurrence of the lesion.

## 3. Discussion

Benign mesenchymoma is a rare soft tissue tumor that consists of fibrous tissue and two or more differentiated types of cells with mesenchyme origin that are not found together at the host site [1]. It is suggested that the tumors may arise due to proliferation of pluripotent primitive mesenchyme cells that undergo differentiation into various elements [1]. They are most commonly found in the upper and lower extremities, kidneys, trunks, and perirenal areas. The majority of them are found in the first decade of life. When analyzing the type of tumor present, it is important to consider the location of the mass and the morphology of cells present. Employing these features allows a tumor of mesenchyme origin to be differentiated from other masses such as hamartomas, choristomas, and teratomas. Hamartoma is a benign malformation that is composed of tissue elements that are disorganized but still resemble the tissue in which they are found. A choristoma is a collection of cells that are microscopically normal but found in an abnormal location. A teratoma is a tumor with components that arise from more than one germ layer that is often present at birth.

Bures and Barnes [2] reported 18 cases of benign mesenchymoma that varied in location from the tongue, epiglottis, trachea, esophagus, and the subcutaneous layer of the skin. Fronie et al. [3] analyzed 167 benign pediatric head and neck tumors with only 16 of them being of mesenchymal origin. Fifteen of these cases were intraoral and the majority of them occurred on the tongue. Yencha et al. [4] identified 36 cases of benign mesenchymomas of the head and neck.

Bures and Barnes [2] stated that, due to benign nature of the tumors, surgical excision is usually curative. However, Le Ber and Stout [1] found a recurrence rate of 20% after surgery. This may be due to the fact that all benign mesenchymomas have microscopic infiltrates into the surrounding tissue to some degree and meticulous surgery with removal of all suspicious tissues is required. There is no malignant potential of these tumors.

CT scan is usually required to define the extent of the lesion and determine its relationship to the important structures in the head and neck. Typically, the lesion appears well defined, with predominance of soft tissue, adipose tissue, and bony or hyaline components [5]. Absence of bone involvement and preservation of adjacent tissue planes are typical of benign pathology.

Surgical excision is usually curative. It is important to excise the lesion completely with a small amount of surrounding normal tissue to prevent recurrence.

## 4. Conclusion

We report a rare case of benign mesenchymoma in the neck of a child closely related to the SM gland. This case is unique, the occurrence of the tumor in this location has not been reported before, and surgical excision with follow-up proved successful. Further research is required on long-term follow-up to determine the behavior patterns, recurrence, and optimal therapy for this rare lesion. Physical exam and imaging techniques are adjunctive to surgical excision and pathological diagnosis.
